# A Role of U12 Intron in Proper Pre-mRNA Splicing of Plant *Cap Binding Protein 20* Genes

**DOI:** 10.3389/fpls.2018.00475

**Published:** 2018-04-16

**Authors:** Marcin Pieczynski, Katarzyna Kruszka, Dawid Bielewicz, Jakub Dolata, Michal Szczesniak, Wojciech Karlowski, Artur Jarmolowski, Zofia Szweykowska-Kulinska

**Affiliations:** ^1^Department of Gene Expression, Institute of Molecular Biology and Biotechnology, Faculty of Biology, Adam Mickiewicz University in Poznan, Poznan, Poland; ^2^Department of Integrative Genomics, Institute of Anthropology, Faculty of Biology, Adam Mickiewicz University in Poznan, Poznan, Poland; ^3^Department of Computational Biology, Institute of Molecular Biology and Biotechnology, Faculty of Biology, Adam Mickiewicz University in Poznan, Poznan, Poland

**Keywords:** U12 introns, U2 introns, mRNA splicing, CBP20, *Arabidopsis thaliana*

## Abstract

The nuclear cap-binding complex (CBC) is composed of two cap-binding proteins: CBP20 and CBP80. The *CBP20* gene structure is highly conserved across land plant species. All studied *CBP20* genes contain eight exons and seven introns, with the fourth intron belonging to the U12 class. This highly conserved U12 intron always divides the plant *CBP20* gene into two parts: one part encodes the core domain containing the RNA binding domain (RBD), and the second part encodes the tail domain with a nuclear localization signal (NLS). In this study, we investigate the importance of the U12 intron in the *Arabidopsis thaliana CBP20* gene by moving it to different intron locations of the gene. Relocation of the U12 intron resulted in a significant decrease in the U12 intron splicing efficiency and the accumulation of wrongly processed transcripts. These results suggest that moving the U12 intron to any other position of the *A. thaliana CBP20* gene disturbs splicing, leading to substantial downregulation of the level of properly spliced mRNA and CBP20 protein. Moreover, the replacement of the U12 intron with a U2 intron leads to undesired alternative splicing events, indicating that the proper localization of the U12 intron in the *CBP20* gene secures correct *CBP20* pre-mRNA maturation and CBP20 protein levels in a plant. Surprisingly, our results also show that the efficiency of U12 splicing depends on intron length. In conclusion, our study emphasizes the importance of proper U12 intron localization in plant *CBP20* genes for correct pre-mRNA processing.

## Introduction

The cap-binding complex (CBC) is a nuclear heterodimer composed of two cap-binding proteins: cap-binding protein 20 (CBP20) and cap-binding protein 80 (CBP80, also known as ABA Hypersensitive 1, ABH1), which binds to the 5′ cap of all RNA polymerase II transcripts. In plants, CBC is important for pre-mRNA and pri-miRNA first intron splicing and regulation of pre-mRNA alternative splicing (Szarzynska et al., 2009; Raczynska et al., 2010). The CBC is also involved in miRNA biogenesis (Kim et al., 2008; Laubinger et al., 2008; Ren and Yu, 2012). Amino acid sequence comparison of *Arabidopsis thaliana* and *Oryza sativa* CBP20 and CBP80 proteins revealed high sequence conservation of the small subunit of CBC (CBP20) and a considerably lower level of conservation of its large subunit (CBP80) (Kmieciak et al., 2002). Interestingly, in comparison to animal CBP20 proteins, plant CBP20s contain an additional carboxy-terminal fragment. The structure of the CBP20 protein in higher plants can be divided into two parts. The core part, built mostly of 138 amino acids, contains the conservative RNA binding domain (RBD), which plays a crucial role in cap structure recognition and binding (Izaurralde et al., 1994), and the tail part, built of approximately 120 amino acids, contains a nuclear localization signal (NLS). In animals, this is the CBP80 protein that carries a functional NLS and is responsible for the import of CBC into the nucleus (Dias et al., 2009). In *A. thaliana*, two functionally independent NLSs are located in the tail part of the CBP20 protein (Kmieciak et al., 2002). In contrast, plants CBP80s do not contain any NLS; thus, the CBP20 protein targets the whole plant CBC to the nucleus. Moreover, in *A. thaliana*, the CBP20 protein is stabilized by CBP80 (Kierzkowski et al., 2009). The down-regulation of nuclear cap-binding proteins in *Arabidopsis* leads to mild developmental abnormalities, such as serrated rosette leaves and delayed development. Interestingly, loss of CBC functions in *Arabidopsis* plants confers hypersensitivity to abscisic acid (ABA) during seeds germination, significant reduction of stomatal conductance, and in consequence greatly enhances tolerance of the *cbp20* and *cbp80* mutants to drought (Hugouvieux et al., 2001; Papp et al., 2004; Jäger et al., 2011; Pieczynski et al., 2013) and salinity (Kong et al., 2014).

*Arabidopsis* and rice *CBP20* gene structures are conserved and contain eight exons. The core part of the CBP20 protein is encoded by the first four exons of the gene, while the tail part is encoded by the last four exons. Six out of seven introns of the *CBP20* gene belong to the classical and abundant U2 introns, while intron no. 4 represents the much rarer U12 introns. Computational analyses allowed the identification in the *A. thaliana* genome 246 U12 introns constituting 0.17% of all predicted introns in this species (Alioto, 2007). Transcriptome analyses, however, identified about eight times more U12 introns (2069 vs. 246) than previously found using the computational approach (Marquez et al., 2012). The majority of U12 introns (89.4%) contain GT-AG terminal dinucleotides; however, a small portion (10.6%) is characterized by the presence of other terminal dinucleotides, of which AT-AC comprises almost half of such non-GT-AG introns (4.8%) (Marquez et al., 2012). U12 introns contain a very characteristic and highly conserved branch point region (TTCCTTRAY), and unlike U2 introns, they do not have any polypyrimidine tract (Lewandowska et al., 2004; Simpson and Brown, 2008; Marquez et al., 2012; Turunen et al., 2013). It has been shown that several proteins of U11/U12 di-snRNP are indispensable for the correct splicing of the U12 intron-containing genes, which is crucial for the normal development of *A. thaliana* (Kim et al., 2010; Kwak et al., 2012; Jung and Kang, 2014; Xu et al., 2016). Moreover, the *Arabidopsis quatre-quart1* (*QQT1*) gene is an indispensable U12 intron-containing gene whose correct splicing is necessary for the wild type phenotype and development of *Arabidopsis* plants (Kwak et al., 2017).

The phylogenetic distribution of U12 introns shows that the minor (U12) splicing pathway appeared very early in eukaryotic evolution, but during the course of evolution, most U12 introns systematically changed to U2 introns (Bartschat and Samuelsson, 2010; Lin et al., 2010). Despite this process, a few U12 introns were retained in selected genes and remain very stable in some taxa (Basu et al., 2008). *CBP20* genes from both *Arabidopsis* and rice were identified as belonging to this small number of genes containing U12 introns with AT-AC terminal dinucleotides. Moreover, the U12 intron in *CBP20* gene in both plant species is located between exons no. 4 and no. 5, splitting the *CBP20* coding sequence into the core and tail CBP20 protein parts in both species studied. Surprisingly, the exons encoding the core part of the protein have identical lengths (18t, 224, 139, and 34 nt), but the exons encoding the tail part of CBP20s differ considerably in length.

In this paper, we show that the *CBP20* gene structure is conserved across land plant species from liverworts to higher plants. All studied *CBP20* genes contain eight exons and seven introns, with the fourth intron belonging to the U12 class. In addition, the length of the first four exons is conserved, while the length of the exons encoding the CBP20 tail part varies considerably in all plant species studied. Furthermore, we show that the *CBP20* U12 introns in plants may be as short as 76 nt and as long as 2733 nt. Our experiments demonstrate that the efficiency of U12 splicing depends on intron length. The experiments carried out to explain the conserved localization of the *CBP20* U12 intron show that the exchange of the *CBP20* U12 intron with the U2 intron leads to undesired alternative splicing events and that the proper localization of the U12 intron in the *CBP20* gene secures correct *CBP20* pre-mRNA maturation and CBP20 protein levels in a plant.

## Materials and Methods

### Plant Material and Growth Conditions

The experiments were performed using *A. thaliana* (L.) Columbia-0 wt plants (Lehle Seeds, Round Rock, TX, United States) and a homozygous T-DNA insertion line *cbp20* (Papp et al., 2004). *Solanum tuberosum* ssp. *tuberosum* var. Sante, *Nicotiana tabacum* var. Xanthi, *Hordeum vulgare* var. Sebastian and liverwort *Pellia endiviifolia* sp B were used for the *CBP20* gene sequencing.

*Arabidopsis* plants were grown in ‘Jiffy-7 42mm’ soil (Jiffy Products International BV, Moerdijk, Nederland) in an MLR-350H Versatile Environmental Test Chamber (Sanyo, Loughborough, Leicestershire, United Kingdom) with a 16 h light/8 h dark photoperiod (approx. 80 μmol m^-2^s^-1^), constant temperature of 22°C and humidity of 70%. Potato plants were grown in sterile conditions in a greenhouse (22°C with constant light, approximately 80 μmol m^-2^s^-1^) on ½ Murashige-Skoog medium pH 5.5–5.6. *N. tabacum* and *H. vulgare* plants were grown in a greenhouse (22°C with 12 h light/12 h dark photoperiod, approx. 120 μmol m^-2^s^-1^) on soil irrigated with mineral nutrients. *P. endiviifolia* sp B was grown as described by Sierocka et al. (2011).

*Arabidopsis thaliana* transformation was performed using the floral-dip method according to Clough and Bent (1998).

### DNA and RNA Isolation

Total genomic DNA, RNA and plasmid DNA were isolated using a DNeasy Plant Mini Kit (Qiagen), RNeasy Plant Mini Kit (Qiagen), and QIAprep Spin Miniprep Kit, respectively, according to protocols supplied by the manufacturers. Purity and amounts of DNA and RNA were determined using NanoDrop Spectrophotometer (Thermo Scientific).

### DNA Sequencing

DNA sequencing was performed with a BigDye v3.1 sequencing kit (Applied Biosystems, Foster City, CA, United States) on a ABI Prism 3130XL Analyzer (Applied Biosystems) in the Molecular Biology Techniques Laboratory, Faculty of Biology, Adam Mickiewicz University in Poznan, Poland.

### cDNA Synthesis, PCR and DNA Cloning

Four micrograms of total RNA was reverse-transcribed using Superscript III Reverse Transcriptase (Invitrogen) and oligo(dT)_15_ primer (Novazym). cDNA of *CBP20* genes from potato, tobacco, barley and liverwort was amplified by PCR using primers designed according to ESTs from NCBI database (Benson et al., 2012). In the case of potato, we designed primers according to the EST 706129 sequence. Tobacco *CBP20* gene cDNA was amplified using primers designed according to the contig sequence assembled on the basis of nine different EST sequences (accession numbers: AM815125, CV017334, AM818189, EB679251, EB440185, AM827799, AM808999, EB444475, and EB678046). Barley *CBP20* cDNA was assembled on the basis of the following EST sequences: TC111197 and TC78828 from TIGR database and BU991417, BJ480285, AJ463125, and AJ475973 from NCBI. Primers for cDNA *CBP20* amplification from *P. endiviifolia* were designed according to the results of whole transcriptome sequencing.

PCR was carried out as described in Szarzynska et al. (2009) and Sierocka et al. (2011). Genomic sequence of the *CBP20* gene from potato and tobacco was amplified using the Expand Long Template PCR System (Roche). PCR products were separated on a 1% agarose gel, purified using a QIAquick PCR Purification Kit (Qiagen), cloned into the pGEM-T Easy vector (Promega) and sequenced. Primers used for amplification of genomic and cDNA sequences of the *CBP20* gene from different plant species are shown in Supplementary Table S1. Amplification of CBP20 cDNAs as well as genes from potato, tobacco, barley and liverwort is shown in Supplementary Figure S1.

### Expression Construct Preparation

Mini- and midi-gene constructs numbers 1, 8, and 9 were prepared by PCR amplification of appropriate *A. thaliana* and *Physcomitrella patens CBP20* gene fragments. Mini- and midi-gene constructs numbers 2–7 were prepared as presented in Supplementary Figure S2. Each construct contains exons no. 4 and no. 5 from the *A. thaliana CBP20* gene. Depending on the construct, these two exons are separated by a U12 or U2 intron originating from different plant species. Mini-gene constructs were prepared in 3- or 4-step PCR. In PCR-1, the U2 or U12 intron was amplified using a forward primer containing on its 5′ end 20 nucleotides complementary to exon no. 4. The reverse primer used in PCR-1 contained at its 5′ end 20 nucleotides complementary to exon no. 5. In PCR-2, the sequence of exon no. 5 was amplified using a forward primer complementary to the last 20 nucleotides of the U2 or U12 intron, depending on the individual mini-gene construct. The final mini-gene sequence was amplified in PCR-3, in which PCR-1 and PCR-2 products were used as templates. In mini-gene constructs containing the U12 intron derived from the *S. tuberosum* or *Vitis vinifera CBP20* gene, an additional PCR-0 was performed in which the U12 intron sequence was amplified.

Each mini-gene construct was cloned into the pDH515 vector at a unique BamHI restriction site within an intronless zein gene encoding a maize seed storage protein. Additionally, the vector contains the 35S promoter of *Cauliflower mosaic virus* (CaMV 35S) and terminator sequence (Lewandowska et al., 2004).

The maxi-gene wt transgene construct was prepared by PCR amplification of the *Arabidopsis CBP20* gene sequence encompassing its native promoter (1228 bp) (Kmieciak et al., 2002). The PCR product was cloned into the NotI restriction site in the pENTR vector. The other five maxi-gene constructs were obtained performing a series of PCRs using the wt transgene construct or *A. thaliana* genomic DNA as a template. Specific sub-fragments of each maxi-gene construct were obtained in the PCR, followed by the consecutive cohesive ends annealing and final PCR amplification (Supplementary Figure S3). The individual maxi-gene constructs were transferred from the pENTR cloning vector to the pEarleyGate302 expression vector using Gateway LR Clonase II Enzyme Mix (Thermo Fisher Scientific). All constructs were confirmed by DNA sequencing. Primers used for construct preparation are listed in Supplementary Table S1.

### Protoplast Isolation, Transfection and Splicing Analysis

Splicing of mini- and midi-gene constructs was analyzed in protoplasts isolated from leaves of *N. tabacum* var. Xanthi (Lewandowska et al., 2004). Protoplast suspension was transfected with each mini- or midi-gene construct as described by Lewandowska et al. (2004). After overnight incubation, total RNA from protoplasts was isolated. cDNA template was synthesized using the zein3′-R reverse primer complementary to the zein sequence flanking mini- and midi-gene constructs in the pDH515 vector (Simpson and Filipowicz, 1996). Splicing analyses were carried out by RT-PCR using following primers: zeinF-FAM labeled with fluorescent phosphoramidite 6-FAM at 5′ end and zeinR (see Supplementary Table S1).

6-FAM-labeled RT-PCR products were quantitatively analyzed using capillary electrophoresis on an ABI 3130xl Genetic Analyzer. The length of the labeled products was calculated using Peak Scanner Software v1.0 (Applied Biosystems) by comparison with the GeneScan-350 TAMRA size standards (Applied Biosystems). Quantification of RT-PCR products was carried out after 23rd amplification cycle by measurement of the fluorescent peak areas of the detected fragments. RT-PCR was carried out in three biological replicates. The same RT-PCR products were separated in parallel on a 1% agarose gel. 6-FAM-labeled fragments were eluted from the gel, cloned into the pGEM T-Easy (Promega) vector and sequenced to confirm specific splicing products.

Splicing analysis of maxi-gene transcripts that were introduced into the *A. thaliana cbp20* mutant was also carried out using capillary electrophoresis, as described above. Primers used for RT-PCR amplification hybridized to the sequence of exons no. 3 and no. 6 of the *CBP20* gene transcript (Supplementary Table S1). The forward primer Splice-FAM-F was labeled at its 5′ end with the fluorescent phosphoramidite 6-FAM.

### RT-qPCR

Real-time RT-qPCR was performed with Power SYBR^®^ Green PCR Master MIX (Applied Biosystems, Warrington, United Kingdom) on a 7900HT Fast Real-Time PCR System (Applied Biosystems) in 10-μl reaction volumes in 384-well plates. To assess the splicing efficiency of mini-genes, an absolute quantification approach was applied. Standard curves were prepared using plasmids containing individual mini-genes. Splicing efficiency was calculated by comparison of spliced and unspliced transcript levels to the total transcript amount. To estimate the total level of the *CBP20* transcript in maxi-gene transgenic lines, the fold change was calculated using the 2-ΔΔCt method. The mRNA fragment of elongation factor 1-alpha (EF1-alpha, TAIR locus: At1g07930) was amplified as a reference gene in *A. thaliana*.

### Western Blot

Protein extracts were separated by 10% SDS-PAGE, transferred to a polyvinylidene fluoride membrane (PVDF; Immobilon^®^-P, Millipore), and analyzed by western blot using antibodies at the indicated dilutions: anti-Actin (691001; MP Biomedicals) at 1:5000 and anti-CBP20 (AS09 530; Agrisera) at 1:1000.

### Bioinformatics Tools

Comparison between DNA and protein sequences was carried out using bioinformatics tools: ClustalW2^1^ and BoxShade^2^. Sequencing results were assembled together using the ContigExpress program from Vector NTI (Invitrogen).

### Sequence Accession Numbers

*Arabidopsis thaliana*, Gene ID: 834443; *Populus trichocarpa*, Gene ID: 7469563; *V. vinifera*, Gene ID: 100265980; *S. tuberosum*, GU046516.1; *N. tabacum*, GU058037.1; *O. sativa*, Gene ID: 4329963; *H. vulgare*, FJ548567.1; *S. moellendorffii* – *Selaginella moellendorffii* v1.0, Scaffold 3333196:1^3^; *P. patens* – *P. patens* V1.2_genome scaffold_105^4^.

## Results

### Plant *CBP20* Gene Structure Is Evolutionarily Conserved and Reveals Preserved Localization of a U12 Intron

**FIGURE 1:**
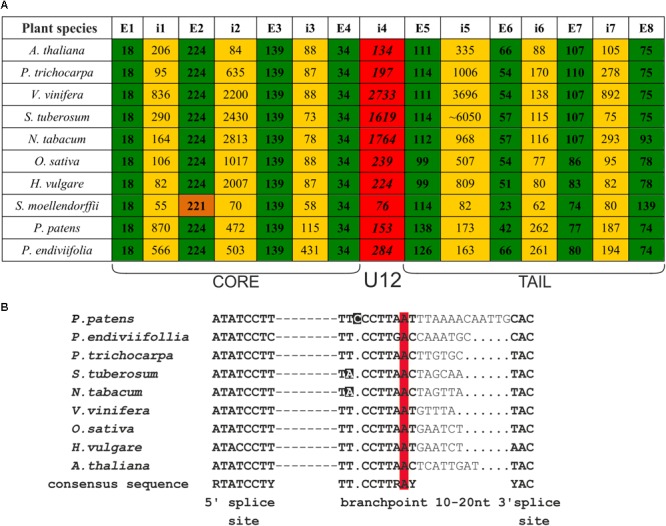
*CBP20* genes derived from different plant species present very similar gene structures and conserved localization of U12 introns. **(A)** Comparison of *CBP20* gene structures. Green color depicts exons, yellow color marks U2 introns, red color indicates U12 introns, and orange color depicts the second exon of the *CBP20* gene from *S. moellendorffii*, which is shorter than other exons in the same position in other plants studied by 3 nucleotides. Note that with the exception of *S. moellendorffii*, the exon lengths are the same in all plant species studied until exon no. 5 is reached, which is separated from the protein core-encoding part of the gene by the U12 intron. E – exon, i – intron. **(B)** A comparison of consensus elements of *CBP20* U12 introns derived from the plant species studied (Simpson and Brown, 2008). Dashed lines represent individual nucleotide sequences between the 5′ splice site and branch point. Dots mark alignment gaps. Nucleotides that differ from the consensus sequence in the branch point region are marked in black. The conserved adenine nucleotides in the branch point are marked in red.

The analysis of *CBP20* genes from *A. thaliana* and *O. sativa* revealed high sequence and structural conservation. To gain deeper insight into the evolutionary conservation of the *CBP20* gene, we decided to compare *CBP20* sequences from various plant species across the plant kingdom. The *CBP20* gene and cDNA sequences from *P. trichocarpa, V. vinifera, Selaginella moellendorffii*, and *P. patens* were found in publicly available databases (**Figure 1**). In addition, full-length *CBP20* cDNA sequences from *S. tuberosum* var. Sante, *N. tabacum* var. Xanthi, *H. vulgare* var. Sebastian and the liverwort *P. endiviifolia* subspecies B were established in our laboratory. Based on the obtained *CBP20* cDNA sequences, we determined the full-length *CBP20* gene sequences in potato, tobacco, barley and liverwort genomes.

We compared the *CBP20* gene structure from ten plant species, including representatives of Bryophyta (liverworts, mosses), Leucophyta (lycophyte), and Spermatophyta (monocots and dicots) (**Figure 1A**). In all species analyzed, the *CBP20* genes contain 8 exons and 7 introns. Interestingly, their first four exons, coding for the core part of the protein, are of the same length with one exception, *S. moellendorffii*, whose second exon is three nucleotides shorter than that in the other *CBP20* genes studied. In all *CBP20* genes, a U12 intron was found separating the evolutionarily conserved core part that encodes a canonical RBD from the carboxy-terminal fragment (tail) that encodes the NLS and generally exhibits a much lower degree of evolutionary conservation. All these U12 introns contain the canonical AT-AC terminal dinucleotides and a branch point sequence typical of U12 introns (**Figure 1B**). The *CBP20* U12 intron length varies from 76 nt in *S. moellendorffii* to 2733 nt in *V. vinifera*. In general, plant species can be divided into two classes: those carrying short U12 introns (from 76 to 284 nt) and those carrying long U12 introns (from 1619 to 2733 nt) (**Figure 1A**).

There are considerable differences between different plant *CBP20* gene lengths. The *S. moellendorffii CBP20* gene (1245 bp) is 10 times shorter than that from *S. tuberosum* (11430 bp). In the potato *CBP20* gene, the fifth intron is extremely long, approximately 6050 bp, which largely accounts for the unusual length of this gene. Computational analysis of this intron sequence has revealed that it contains characteristic signatures of the LINE1 retrotransposon. Since we could not identify any full open reading frame for reverse transcriptase, we assumed that this retroelement is defective.

Comparison of all plant CBP20 amino acid sequences revealed the presence of conserved residues within the core domain that were reported to be responsible for binding to the cap structure and for recognition of RNA (Supplementary Figure S4) (Mazza et al., 2002). In the tail fragment of all CBP20 proteins, we identified potential NLS sequences. In the majority of plant species, the *CBP20* gene contains two NLS motives, as was shown before for *A. thaliana*, while in *P. trichocarpa* and *P. patens*, we identified only one motif: the proximal NLS. Generally, sequence homology of full-length CBP20 proteins in plants ranges from 61 to 79% identity, and that of the highly conserved core part ranges from 80 to 89% identity. The homology within the tail part is rather low (39–69% identity), but the distal part of this tail region shows again a relatively high degree of conservation, suggesting that this part of the protein may play a structural or functional role.

It was shown that U12 introns over the course of evolution were usually exchanged with U2 introns (Burge et al., 1998; Bartschat and Samuelsson, 2010). The evolutionarily conserved localization of the plant *CBP20* U12 intron separating the conserved core domain from the tail part of the protein in all *CBP20* plant genes characterized encouraged us to investigate the role of this intron in *CBP20* pre-mRNA maturation.

### Efficiency of U12 Splicing Depends on the Intron Length

**FIGURE 2:**
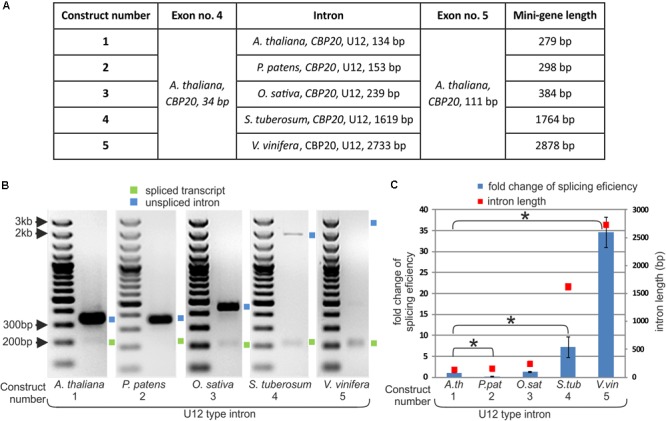
The efficiency of *CBP20* U12 intron splicing depends on intron length. **(A)** A table showing structures of five mini-gene constructs containing exons no. 4 and no. 5 from the *A. thaliana CBP20* gene separated by one of the U12 introns differing in length and derived from different plant species. **(B)** Qualitative RT-PCR analysis of splicing of mini-gene transcripts in tobacco protoplasts. Blue and green squares represent unspliced and spliced transcripts, respectively. **(C)** Quantitative Real-time PCR analysis of mini-gene transcript splicing efficiency. Splicing efficiency of the mini-gene no. 1 transcript carrying the U12 intron from the *Arabidopsis CBP20* gene was taken as 1. Blue bars represent fold change in splicing efficiency in comparison to the mini-gene no. 1 transcript. Red squares show the length of U12 introns derived from various plant species studied. Values are shown as the mean ± SD (*n* = 3) from three independent experiments. Asterisk – *P* < 0.001, Student’s *t*-tests.

As we have shown, the length of *CBP20* U12 introns in the studied plant species varies considerably. To test whether U12 intron length may influence U12 intron splicing efficiency, we prepared five mini-gene constructs. These mini-genes consist of *A. thaliana CBP20* exon no. 4 (34 bp) and exon no. 5 (111 bp) separated by the following: construct no. 1 – the original *A. thaliana CBP20* U12 intron (134 bp), construct no. 2 – the *P. patens CBP20* U12 intron (153 bp), construct no. 3 – the *O. sativa CBP20* U12 intron (239 bp), construct no. 4 –the *S. tuberosum CBP20* U12 intron (1619 bp) and construct no. 5 – the *V. vinifera CBP20* U12 intron (2733 bp) (**Figure 2A**). The mini-genes were cloned into the pDH515 expression vector (Lewandowska et al., 2004) and sequenced. These recombinant plasmids were then used for tobacco mesophyll protoplasts transfection. Total RNA was isolated from the transfected protoplasts incubated overnight, and cDNA derived from the mini-gene transcripts was prepared. Splicing analyses were carried out applying RT-PCR with fluorescent-labeled primers. The splicing efficiency of each construct was determined as the mean of three independent experiments. All U12 introns were spliced correctly; no alternative splicing events were observed (**Figure 2B**). However, the splicing efficiency of different mini-genes varied considerably. We found a correlation between the U12 intron length and splicing efficiency as revealed by RT-qPCR using the absolute quantification method (**Figure 2C** and Supplementary Table S2). The splicing efficiency of the 134 nt long U12 intron from *A. thaliana* was assumed in this comparison to be 1. The U12 introns derived from *O. sativa, S. tuberosum* and *V. vinifera CBP20* genes were spliced 1.28, 7.17, and 34.65 times more efficiently than that from *A. thaliana*, respectively (**Figure 2C**).

An exception to this rule was observed in the splicing efficiency of the U12 intron derived from *P. patens*. Despite its almost identical length with the U12 intron from *A. thaliana*, the splicing efficiency of this U12 intron was five times lower.

### The Replacement of a U12 Intron With a U2 Intron in the *CBP20* Mini-Gene Improves Splicing Efficiency but Leads to Deleterious and Undesired Improper Splicing Events

**FIGURE 3:**
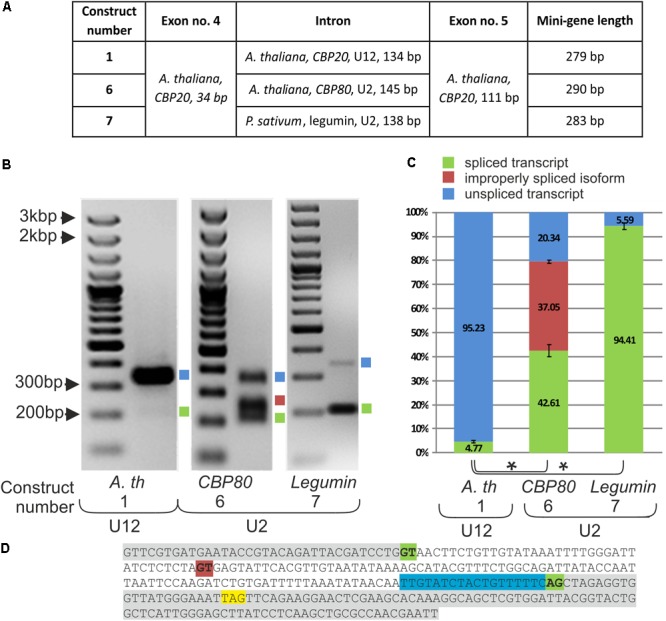
Replacement of the original *CBP20* U12 intron by a U2 intron may lead to deleterious splicing events. **(A)** A table showing structures of three mini-gene constructs containing exons no. 4 and no. 5 from the *A. thaliana CBP20* gene separated by the original U12 intron (construct no. 1), by the U2 intron from the *Arabidopsis CBP80* gene (construct no. 6), and by the U2 intron from the *P. sativum* legumin gene (construct no. 7). **(B)** Qualitative RT-PCR analysis of splicing of mini-gene transcripts in tobacco protoplasts. Blue and green squares represent unspliced and spliced transcripts, respectively. The red square represents the alternatively spliced *CBP80* U2 intron. **(C)** Quantitative analysis of mini-gene transcripts RT-PCR products using capillary electrophoresis. Splicing efficiency is calculated as a percentage of the sum of all detectable products. Values are shown as the mean ± SD (*n* = 3) from three independent experiments. **(D)** Nucleotide sequence of the mini-gene no. 6 containing the *Arabidopsis CBP80* gene U2 intron. Green color depicts constitutive U2 5′ and 3′ splice site dinucleotides, red color represents an alternative 5′ splice site, the STOP codon is marked in yellow, and blue color stands for the polypyrimidine tract. Asterisk – *P* < 0.005, Student’s *t*-tests.

To learn more about the role of the U12 intron in *CBP20* plant genes, we constructed a series of mini-genes containing *A. thaliana* exons no. 4 and no. 5 separated by the original *CBP20* U12 intron (134 bp – construct no. 1), the U2 intron derived from the *A. thaliana CBP80* gene (intron no. 11, 146 bp – construct no. 6) and the U2 intron derived from the *P. sativum* legumin gene (intron no. 1, 138 bp – construct no. 7) (**Figure 3A**). Both U2 introns were chosen because of their similar length to that of the original *A. thaliana CBP20* U12 intron. The selected U2 introns have canonical GT-AG splice site dinucleotides and a polypyrimidine tract near the 3′ splice site and show a low GC content, 26% and 29% for the pea legumin and *Arabidopsis CBP80* genes, respectively. U12 intron from the *A. thaliana CBP20* gene has also a low GG content – 36%.

Splicing of the mini-gene transcripts was studied in the tobacco protoplast system, as described in the case of mini-genes containing various U12 introns. The original U12 intron was spliced with only 4.77% efficiency, whereas both studied U2 introns, from the *Arabidopsis CBP80* gene and pea legumin gene, were spliced, reaching 79.66 and 94.4% efficiency, respectively (**Figure 3B**). These results are supported by data that have been previously obtained in our laboratory (Lewandowska et al., 2004), showing that *A. thaliana* U12 introns are spliced less efficiently than are U2 introns in tobacco protoplasts. However, in the mini-gene containing the U2 intron derived from the *A. thaliana CBP80* gene, an alternative splicing event was observed: two alternative 5′ splice sites were recognized by the tobacco U2 splicing machinery, leading to the proper splicing of the *CBP20* mini-gene transcript (42.61%) or to the inclusion of an additional 38 nt from the 5′ intron end into the spliced mRNA (37.05%) (**Figures 3C,D**). The 38 nt long insertion introduces a premature stop codon resulting in premature translation termination. To test whether this alternative splicing event also occurs naturally in the *CBP80* gene, we analyzed the splicing of this intron in the *CBP80* transcript. We were able to detect only constitutively, properly spliced *CBP80* mRNA, without any additional alternative splicing events (Supplementary Figure S5).

### *Arabidopsis CBP20* U12 Intron Surrounded by Additional Exons and U2 Introns Is Not Efficiently Recognized by the U12 Splicing Machinery

**FIGURE 4:**
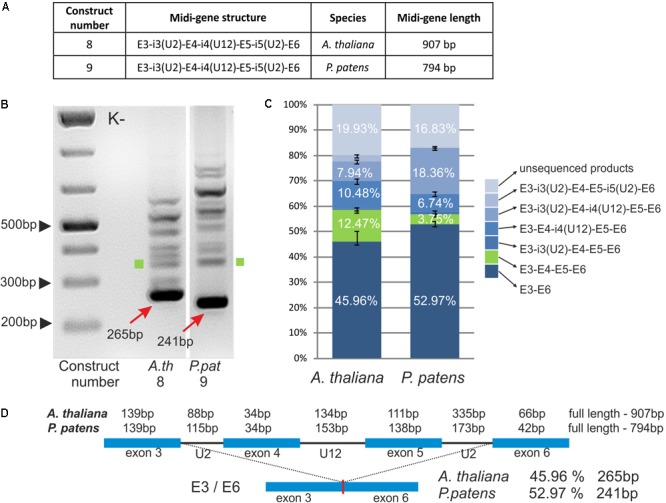
Plant *CBP20* U12 intron surrounded by four exons and two U2 introns is not efficiently recognized by the U12 splicing machinery. **(A)** A table showing structures of two midi-gene constructs containing *A. thaliana* or *P. patens CBP20* gene fragment of the following composition: exon 3 – U2 intron 3 – exon 4 – U12 intron 4 – exon 5 – U2 intron 5 – exon 6. **(B)** Qualitative RT-PCR analysis of splicing of midi-gene transcripts in tobacco protoplasts. Arrows point to the improperly spliced products in which exons no. 4 and no. 5 are skipped. Green squares represent properly spliced midi-gene CBP20 transcripts. The rest of the bands above the main amplification products represent a variety of partially spliced transcripts. **(C)** Quantitative analysis of midi-gene transcript RT-PCR products using capillary electrophoresis. Splicing efficiency is calculated as a percentage of the sum of all detectable products. Values are shown as the mean ± SD (*n* = 3) from three independent experiments. Green color depicts properly spliced *CBP20* mRNA isoform. **(D)** Structure of midi-genes and fully spliced transcript representing the most abundant splicing event.

It has been shown that the extension of mini-genes composed of two exons from *GSH2* interrupted by a U12 intron with neighboring exons and U2 introns improves U12 splicing efficiency (Lewandowska et al., 2004). To test whether this is also true for other genes containing U12 introns, we prepared two midi-genes that were based on *A. thaliana* and *P. patens CBP20* genes. *A. thaliana* (construct no. 8) and *P. patens* (construct no. 9) midi-genes consist of E3-i3(U2)-E4-i4(U12)-E5-i5(U2)-E6 from the *A. thaliana* and *P. patens CBP20* genes, respectively (**Figure 4A**). The midi-genes were studied in tobacco protoplasts as described before in the case of mini-genes.

The splicing patterns of *A. thaliana* and *P. patens* midi-transcripts were similar (**Figure 4B**). We obtained one main and many additional minor products. Sequencing of these products has shown that the main band (265 bp in *A. thaliana* and 241 bp in *P. patens*) represents a spliced product consisting of exons no. 3 and no. 6. Thus, splicing in that case leads to the exon skipping event by the removal of all three introns and exons no. 4 and no. 5. The remaining minor bands represent various spliced products listed in **Figure 4C**, among which a fully and properly spliced transcript containing all four exons (E3-E4-E5-E6) was also detected. Quantitative analysis shows that the main improperly spliced product was present in approximately 50% of all other differentially spliced RNA molecules, in both *Arabidopsis* and *Physcomitrella* midi-genes (**Figures 4C,D**). The fully spliced transcript containing all four exons (E3-E4-E5-E6) was present only in approximately 12.47% of all splicing products in *Arabidopsis* and 3.75% in *Physcomitrella*. Thus, in contrast to the *GSH2* midi-gene, the neighboring U2 introns and exons do not enhance the recognition of the U12 intron in the case of *Arabidopsis* and moss *CBP20* midi-genes. Instead, the U12 intron signals are mainly not recognized properly in these constructs.

### The Proper Localization of the U12 Intron in the *CBP20* Gene Secures Correct Pre-mRNA Maturation

**FIGURE 5:**
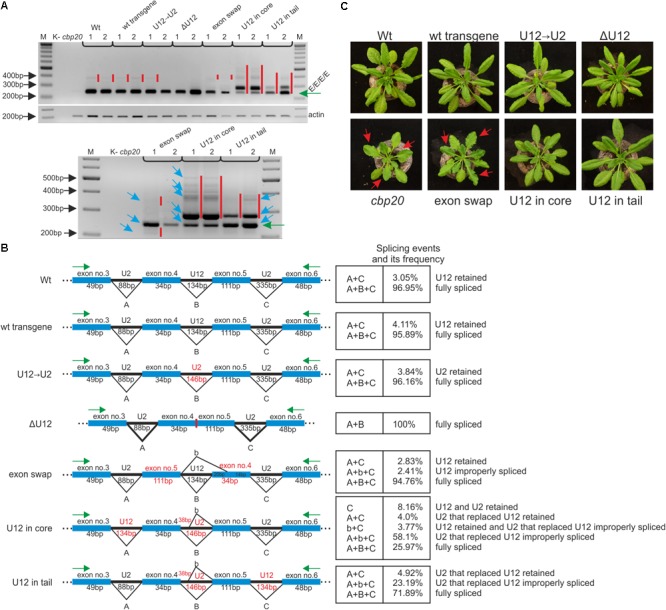
The proper localization of the U12 intron in the *CBP20* gene secures correct pre-mRNA maturation in plant. **(A)** Qualitative RT-PCR analysis of splicing of *CBP20* full-length maxi-gene transcripts. For each construct, two independent transgenic lines were analyzed. Electrophoretic separation of RT-PCR products was carried out in a 1.2% agarose gel. Abbreviations: K- – negative control (no template); *cbp20* – mutant line; Wt – wild-type plants; wt transgene – depicts wild-type *CBP20* gene; U12→U2 – the *CBP20* gene in which the original U12 intron was exchanged by the U2 intron derived from the *Arabidopsis CBP80* gene; ΔU12 – the *CBP20* gene with U12 intron deletion; exon swap – the *CBP20* gene mutant in which exons no. 4 and no. 5 flanking the U12 intron have been exchanged; U12 in core – construct derived from the U12→U2 construct in which the U12 intron has been moved between exons no. 3 and no. 4 (core part of the *CBP20* gene); and U12 in tail – construct derived from the U12→U2 construct in which the U12 intron has been moved between exons no. 5 and no. 6 (tail part of the *CBP20* gene). All constructs are under a native promoter. Green arrow points the properly spliced *CBP20* transcript (E/E/E/E). Red bars show additional detectable partially spliced or improperly spliced products. A panel below shows a zoomed-in fragment of the above gel; blue arrows depict sequenced and quantitatively analyzed products. Actin – loading control. M – molecular DNA marker. **(B)** A scheme presenting all detected *CBP20*-gene derived mutant splicing isoforms. Capital A, B, and C letters represent constitutive splicing events for introns no. 3, no. 4, and no. 5, respectively. b – depicts improper splicing events of intron no. 4. Quantitative analysis of mini-gene transcript RT-PCR products was performed using capillary electrophoresis. Splicing efficiency is calculated as a percentage of the sum of all detectable products. Values are shown as the mean ± SD (*n* = 3) from three independent experiments. **(C)** *Arabidopsis* vegetative rosettes of wt and *CBP20* maxi-gene lines after 30 days of growth.

To study the influence of the U12 intron on the splicing efficiency of the full *CBP20* pre-mRNA in plants, we prepared constructs composed of the natural *A. thaliana CBP20* gene promoter and the whole wild-type (wt) gene sequence or its variants (the maxi-genes series) (**Figure 5B**). In addition to the wt maxi-gene (wt transgene), we constructed *CBP20* gene variants in which (i) the U12 intron was replaced with the U2 intron derived from the *A. thaliana CBP80* gene (intron no. 11; U12→U2), (ii) the U12 intron was removed (ΔU12), and (iii) exons no. 4 and no. 5 were swapped (exon swap). Additionally, we prepared two constructs based on the *CBP20* gene variant U12→U2. In the first construct, the U12 intron was moved upstream in the *CBP20* gene and replaced the U2 intron no. 3 (U12 in core); by this, we moved the U12 intron into the core-encoding part of the *CBP20* gene. In the second construct, the U12 intron replaced the original U2 intron localized between exons no. 5 and no. 6 (U12 in tail); in this construct, the U12 intron was moved into the tail-encoding part of the *CBP20* gene. *A. thaliana cbp20* mutant plants were transformed with these constructs, and three independent homozygous lines of each gene variant were further tested.

Qualitative RT-PCR-based analyses of the *CBP20* mRNA levels revealed the presence of one dominant product representing fully spliced transcripts in the case of wt plants as well as transgenic plants carrying the wt *CBP20* gene construct (wt transgene) and the U12→U2 construct. A small amount of partially unspliced products was also detected (**Figure 5A**, upper panel). In transgenic plants carrying mutated *CBP20* genes in which the U12 intron was deleted (ΔU12), we also observed the presence of fully and properly spliced *CBP20* mRNA. Moreover, the transgenic plants exhibited a wt phenotype, indicating that the CBP20 protein is produced in the transgenic lines analyzed (**Figure 5C**, upper panel).

Only transgenic plants carrying the *CBP20* gene with swapped exons (exon swap) gave mRNAs in which the CBP20 coding sequence was disrupted. This impaired mRNA accumulated at a lower level than in wt and wt transgene plants containing a wt copy of the *CBP20* gene (**Figure 5A**, upper panel). The exon swapping mRNA contains a premature stop codon (in exon no. 5) that might cause the synthesis of putative shorter proteins with a disturbed core fragment. As expected, this mutant plants’ phenotype is very similar to that of the null *cbp20* mutant, exhibiting serrated rosette leaves and growth retardation (**Figure 5C**, lower panel), confirming that CBP20 is indeed not produced in these plants. In the case of mutants in which the U12 intron was moved into the core- or tail-encoding parts of the gene, additional splice products were observed (**Figure 5A**, upper panel; the lower panel represents a zoomed in part of the upper one).

Quantitative RT-qPCR analyses using fluorescent-labeled primers and capillary electrophoresis were carried out to measure the levels of individual spliced products. Cloning and sequencing of these products were performed to identify particular splicing isoforms. In the case of ΔU12, apart from the fully spliced product, we did not detect any additional mRNA isoforms. In the wt, wt transgene and U12→U2 plants, the fully spliced mRNA represented 96–97% of all spliced products, while U12- or U2-containing mRNA isoforms were present in 3–4% of all spliced products (**Figure 5B**). The exchange of exons no. 4 and no. 5 within the *CBP20* gene resulted in the presence of properly spliced mRNA (approximately 95% of all isoforms), the isoform still containing the U12 intron (approximately 3% of all splicing isoforms) and an additional mRNA isoform in which the 3′ alternative U12 splice site was selected within exon no. 4 (approximately 2.5% of all splicing isoforms).

Two *A. thaliana* lines in which the U12 intron was relocated into the core- or tail-encoding part of the *CBP20* gene (U12 in core and U12 in tail) produced complex patterns of mRNA isoforms. In the case of the U12 in core variant, we identified and quantitatively measured the levels of five mRNA isoforms. The fully and correctly spliced *CBP20* mRNA isoform represented only 26% of all splicing products (**Figure 5B**). Surprisingly, the most abundant mRNA isoform showed an improperly spliced U2 intron replacing the original U12 intron. The alternative 5′ splice site was selected, leading to the inclusion of an additional 38 nt from the 5′ intron end into the processed mRNA (58.1%) (**Figure 5B**). However, these transgenic plants exhibit the wt phenotype, suggesting that the lower level of properly spliced *CBP20* mRNA is sufficient to fulfill plant requirements for the CBP20 protein (**Figure 5C**). Interestingly, an identical improper splicing event was observed in the mini-gene containing the same U2 intron instead of the original U12 intron (**Figure 3B**). Three additional mRNA isoforms were observed in small amounts: (1) the isoform in which the U12 intron as well as the U2 intron that replaced the original U12 intron were retained (8.16%), (2) the isoform in which the U2 intron that replaced the original U12 intron was retained (4.0%), and (3) the isoform in which the U12 intron was retained and the U2 intron located between exons no. 3 and no. 4 was incorrectly spliced (3.8%).

In the U12 in tail maxi-gene variant we identified and measured the levels of three mRNA isoforms. The fully and correctly spliced *CBP20* mRNA isoform represented almost 72% of all mRNA isoforms (**Figure 5B**). A minor portion of spliced products were identified as an isoform still containing the U2 intron that replaced the original U12 intron (4.9%). We also detected an mRNA isoform identical to that in the U12 in core maxi-gene, which contained the improperly spliced U2 intron that replaced the original U12 intron (23.2%) (**Figure 5B**). As expected, all these plants exhibit a wt phenotype (**Figure 5C**). These results suggest that shifting the U12 intron into other intron positions in the *Arabidopsis CBP20* gene disturbs splicing events, leading to substantial downregulation of the properly spliced mRNA level.

**FIGURE 6:**
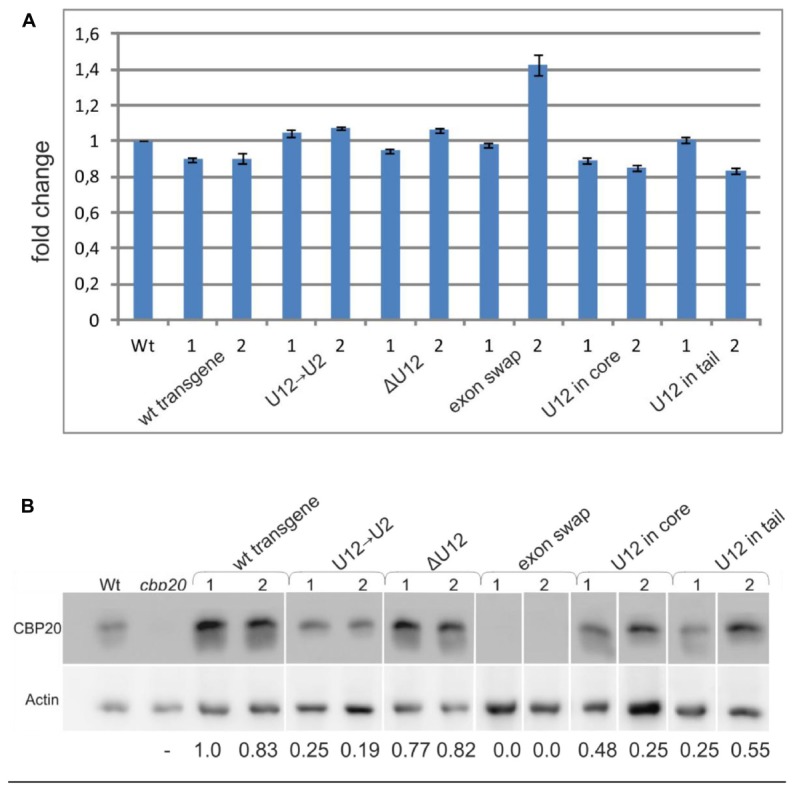
The localization, lack or replacement of the U12 intron in the *CBP20* gene does not influence the total level of the *CBP20* transcript but impacts the level of CBP20 protein in plants. **(A)** Real-time qPCR analysis of the total level of the *CBP20* transcript in maxi-gene transgenic lines. For each construct, two independent transgenic lines were analyzed. Wt – wild-type plants; *cbp20* – *cbp20* mutant line; wt transgene – wild-type *CBP20* gene structure; U12→U2 – *CBP20* gene in which the original U12 intron was replaced with the U2 intron derived from the *Arabidopsis CBP80* gene; ΔU12 – *CBP20* gene with U12 intron deletion; exon swap – the *CBP20* gene in which exons no. 4 and no. 5 flanking the U12 intron have been exchanged; U12 in core - a derivative of the U12→U2 construct in which the U12 intron has been introduced between exons no. 3 and no. 4; and U12 in tail - derivative of the U12→U2 construct in which the U12 intron has been introduced between exons no. 5 and no. 6. The *CBP20* mRNA level in Wt was taken as 1. Values are shown as the mean ± SD (*n* = 3) from three independent experiments. **(B)** Western blot analysis of CBP20 protein levels in transgenic *Arabidopsis* plants. For each construct, two independent transgenic lines were analyzed. Upper panel – immunoblot using antibodies against CBP20 protein; lower panel – immunoblot with antibodies against actin used as a loading control. Numbers below the western blot image are relative intensities of CBP20 bands calculated using the wt transgene (line 1) CBP20 level as 1. Lines are described as previously. The western blot representing recognition of CBP20 in wild type and various mutants of this gene in *A. thaliana* is fused because the original blots contained data from additional transgenic lines, not included in the paper. The data are from two independent western blots. All calculations concerning the amount of the CBP20 protein were carried out separately for each western.

To quantify the total level of *CBP20* transcript in the wt and maxi-gene transgenic lines analyzed, we performed real-time RT-PCR using primers designed to recognize the 3′ ends of the transcripts. No significant differences in the *CBP20* mRNA levels between wt and transgenic plants were observed (**Figure 6A**). However, we did detect changes between maxi-gene transgenic lines when the CBP20 protein level was analyzed by western blotting (**Figure 6B**). A similar protein level was observed for the wt transgene and ΔU12 lines. As expected, no CBP20 protein was detected in the exon swapping maxi-gene line due to the premature STOP codon existing in exon no. 5 of this construct. Interestingly, the decreased level of CBP20 was observed in the U12→U2 transgenic line as well as in the U12 in core and U12 in tail transgenic lines.

## Discussion

The evolutionary conservation from liverworts to higher plants of the U12 intron localization within the plant *CBP20* genes encouraged us to study the role of this intron in the *CBP20* pre-mRNA maturation. It has been reported that U12 intron positions are more strongly conserved between animals and plants than are the positions of U2 introns (Basu et al., 2008). Similar to plant *CBP20* genes, human, chicken, and starlet sea anemone *CBP20* genes also contain U12 introns. However, the animal *CBP20* gene structures and positions of U12 introns differ significantly from those of plant *CBP20* genes (Supplementary Figure S6). In contrast to their animal orthologs, plant *CBP20* genes contain an additional coding sequence representing the so-called tail part of CBP20. In each plant *CBP20* gene, a U12 intron separates the core part of the protein from the tail fragment. The tail part of plant CBP20s usually contains two NLSs, which is characteristic of plant small CBC subunits. Our *in silico* analyses revealed, however, that the *Chlamydomonas reinhardtii CBP20* gene does not have the tail-encoding part (GenBank: EDP01640.1). Interestingly, the *Chlamydomonas CBP20* gene also does not contain a U12 intron. Moreover, we have not found U12 introns in fungal *CBP20* genes that also lack the plant-specific CBP20 tail. Therefore, it can be speculated that the U12 intron in *CBP20* genes appeared during land plant evolution and was introduced into the gene coding sequence together with the tail-encoding part of *CBP20*.

Comparison of the *CBP20* U12 introns of various plants revealed their broad spectrum of length: they can be as short as 76 bp (*S. moellendorffii*) and as long as 2733 bp (*V. vinifera*). Browsing the U12 database (U12DB) for U12 introns present in the *A. thaliana* genome revealed the presence of 246 U12 introns (Alioto, 2007). The length of these introns varies between 68 and 3731 bp. However, the number of U12 introns exceeding 1000 bp in length is rather low (only 6 such introns were described). The number of *Arabidopsis* U12 introns presented in the U12DB is underestimated, because more recent studies based on transcriptomic data revealed 2069 U12 introns in *Arabidopsis* (Marquez et al., 2012). Our data show that plant *CBP20* U12 introns derived from different plant species, when inserted into the mini-genes containing two flanking *A. thaliana* exons, exhibit differential impact on their splicing efficiency. Surprisingly, the longer the U12 intron was, the higher splicing efficiency was observed. We have previously reported that the splicing efficiency of plant U12 introns depends on a combination of factors, including TA content, exon splicing enhancer sequences (ESEs), and the presence of an adenosine at the upstream purine position in the plant U12 branch point consensus TCCTTRATY (Lewandowska et al., 2004). All *CBP20* U12 introns studied in this paper exhibit a similar high TA content (approximately 65%), and almost all (except *P. endiviifolia*) contain an adenosine at the upstream position in their branch point sequences (**Figure 1**). Since the flanking exons are identical in all constructs, one can suggest that putative ESEs should influence all studied U12 intron splicing in the same way. Thus, the only evident difference between short and long *CBP20* U12 introns is that the longer U12 introns contain more TA-rich sequences, which may help in recognition of these introns by the U12 splicing machinery. Therefore, it is possible that one of the yet-unidentified hnRNP proteins recognizing TA-rich sequences within U12 introns plays an important role in plant minor spliceosome recruitment.

We noticed that the *CBP20* U12 intron from *P. patens* was extremely inefficiently spliced in tobacco protoplasts (**Figure 2**). Previously we also observed very low splicing efficiency of mini-genes containing U12 introns originated from different plant genes (Lewandowska et al., 2004). We showed that U12 intron splicing was enhanced by increased TA-richness of the intron. In this study we found that the branch point of the *P. patens CBP20* U12 intron has an additional C nucleotide in the 5′ part of the consensus sequence compared to the *CBP20* U12 introns from other plant species (**Figure 1**). We analyzed all U12 introns from the *P. patens* genome and compared their branch points to the consensus sequence. Only four *P. patens* U12 introns containing the additional C nucleotide in the 5′ part of the branch point were found (124 *P. patens* U12 introns were analyzed). Therefore, the low efficiency of *P. patens CBP20* U12 intron splicing could be due to the specific sequence of its branch point. Searching the U12DB for this additional C in the *A. thaliana* 5′ part of all U12 branch point sequences revealed the presence of only six such examples (Alioto, 2007). This shows that the presence of an additional cytidine in the branch point sequence is acceptable for both *Physcomitrella* and *Arabidopsis* U12 splicing machineries but may be a reason for the inefficient splicing of introns containing such branch point sequence. Further investigations are needed to uncover the role of this additional C in plant U12 intron splicing. On the other hand, the low splicing efficiency of the *P. patens CBP20* U12 intron may also be associated with the compatibility of specific intron/exon sequences as well as ESEs that are present or absent in different exonic sequences. Comparison of *P. patens* and other plant species exon sequences that surround *CBP20* U12 introns revealed a high similarity between exon no. 4, but in the case of exon no. 5, four insertions (3, 6, 14, and 5 bp long) and a single nucleotide deletion were found in *P. patents* (Supplementary Figure S7). Therefore, we cannot exclude the possibility that these exon differences between *Arabidopsis* and *Physcomitrella* may cause the incompatibility in the proper recognition of *P. patens* U12 intron-containing mini-gene transcripts by the tobacco U12 minor spliceosome.

To explain a role of the U12 intron in *CBP20* pre-mRNA maturation, several mini-, midi- and maxi-gene constructs were prepared. This approach allowed us to dissect the effect of given CBP20 transcript fragments on U12 intron splicing. The shortest constructs (the mini-genes series) contained two *CBP20* exons that originally flank the U12 intron. The U12-containing mini-transcripts were correctly but poorly spliced (4.77%). In a similar assay, the pea U2 legumin intron was spliced correctly and very efficiently (94.41%). The *CBP80* U2 intron was spliced more efficiently (42.61%) than the original U12 intron but less efficiently than the legumin gene-derived U2 intron. In addition, alternatively spliced products were frequently observed (37.05%) (**Figure 3C**). These results have shown that the replacement of the U12 intron with a U2 intron increases splicing efficiency but may lead to undesired improper splicing events. Our observation agrees with the published data showing that U12 splicing slows down pre-mRNA processing (Lewandowska et al., 2004; Simpson and Brown, 2008). We demonstrated, however, that U2 introns when inserted between exons normally flanking a U12 intron are spliced improperly, which may lead to the downregulation of CBP20 protein expression (**Figure 4**). It has been shown in animal cells that U12 intron splicing is a limiting step in pre-mRNA processing (Patel et al., 2002; Niemelä and Frilander, 2014).

Since the *CBP20* U12 intron splicing efficiency of the *Arabidopsis* mini-gene transcript (construct no. 1, **Figure 2**) was very low (0.34%), we decided to extend the construct, and we modified it by the introduction of two U2 introns and two flanking exons of the *Arabidopsis CBP20* gene (the midi-gene series). Indeed, we improved the proper splicing efficiency almost three times (from 4.77 to 12.47%, **Figure 4**). Unexpectedly, this correct splicing was predominated by an extensive skipping event resulting in the production of fused exons no. 3 and no. 6 (approximately 50% of all spliced products). In addition, a plethora of minor alternatively spliced products were observed. A similar effect was observed in the case of midi-gene transcripts containing the *Physcomitrella CBP20* intron, suggesting the general character of the results obtained (**Figure 4**). In our earlier studies, we used a similar approach to study analogous mini- and midi-gene constructs of the *Arabidopsis GSH2* gene containing a U12 intron (Lewandowska et al., 2004). Unlike the results of the experiment presented in this paper, in the case of *GSH2*, we detected only constitutive splicing events, and as a result, we obtained the fully spliced products consisting of two or four exons when the mini- and midi-genes were analyzed, respectively. In addition, the transcript derived from the midi-gene (containing additional exon and intron sequences that flanked the U12 intron) was spliced more efficiently. This suggested that the U2 introns located upstream and downstream of U12 participated in the mechanism of definition of the U12 intron (Lewandowska et al., 2004). Interestingly, overexpression of the RBP45 protein, an hnRNP-like RNA binding protein with affinity for U-rich sequences (Lorković et al., 2000), resulted in a complete abolishment of the normal splicing pattern of the *GSH2* midi-gene transcript. RBP45 increased the overall splicing efficiency of the transcript, but caused an extensive skipping event resulting in the accumulation of RNA molecules that were composed of fused two terminal exons (Lewandowska et al., 2004). In the majority of *CBP20* midi-gene constructs analyzed in this paper, the presence of the U12 intron was ignored, and the most proximal 5′ as well as distal 3′ U2 splice sites were predominantly selected, without overexpression of RBP45. This can suggest that within the *CBP20* midi-gene transcripts are sequences with high affinity to RBP45 or other yet-unidentified tobacco RBP45-like proteins that can stimulate selection of the most external 5′ and 3′ splice sites.

Interestingly, the skipping effects that were observed during splicing of the midi-gene constructs were not detected when full-length *CBP20* gene variants (the maxi-genes series) were tested in transgenic plants. The wt transgene transcripts were correctly spliced, and removing the U12 intron (the ΔU12 *CBP20* gene version) did not change the correct splicing pattern. The splicing pattern was changed, however, when the position of the U12 was altered (moved to the core or tail part of the *CBP20* gene), and a U2 intron replaced the original *Arabidopsis* U12 intron. In transgenic lines expressing these two constructs, incorrect splicing of the U2 intron, which served in the tested gene variants as intron no. 4, was detected. This is interesting since the same intron surrounded only by two U2 introns (a midi-gene) was spliced correctly, without any alternative events. Thus, in context of the whole *CBP20* gene, the U12 intron present upstream or downstream of its natural location induced selection of alternative splice sites. Moreover, the exon-swapping version of the *CBP20* gene (exons no. 4 and no. 5 were swapped) caused alterations in splice site selection of the U12 intron. These results show that sequences of both exons no. 4 and no. 5, as well as location of the U12 intron, are necessary for the proper maturation of *A. thaliana CBP20* pre-mRNA. However, in all maxi-gene constructs analyzed, the levels of their transcripts were comparable to those of the wt (**Figure 6A**), suggesting that the alternative splicing events detected in some *CBP20* gene variants do not dramatically influence the levels of primary transcripts produced from the transgenes used in this study. Surprisingly, the protein level of CBP20 originating from the U12→U2 construct was lower than that expressed from the wt transgene. This low level of CBP20 was observed in two independent transgenic lines. Less CBP20 was also observed in the U12 in core and U12 in tail transgenes, and as expected, no CBP20 was detected in the exon swapping. These differences in the levels of CBP20, which are not supported by alterations in *CBP20* gene expression at the mRNA level, suggest that mature mRNAs originating from some of our constructs are partially retained in the nucleus and are not exported to the cytoplasm to be used as templates in translation. Further studies must be carried out to fully uncover the connection between U12 intron splicing, mRNA export and translation in the cytoplasm.

## Author Contributions

MP and KK designed and performed the experiments, analyzed the data, and wrote the manuscript. DB and JD performed the experiments and contributed to data analysis. MS and WK conducted the bioinformatic analyses. AJ advised on experiments and data analysis and assisted in drafting the manuscript. ZS-K conceived the study contributed to data interpretation and participated in the manuscript writing. All authors read and approved the final manuscript.

## Conflict of Interest Statement

The authors declare that the research was conducted in the absence of any commercial or financial relationships that could be construed as a potential conflict of interest.

## Footnotes

1 www.ebi.ac.uk

2 www.ch.embnet.org

3 https://genome.jgi.doe.gov

4 www.cosmoss.org
