# Corrigendum: A Role of U12 Intron in Proper Pre-mRNA Splicing of Plant *Cap Binding Protein 20* Genes

**DOI:** 10.3389/fpls.2019.01287

**Published:** 2019-10-11

**Authors:** Marcin Pieczynski, Katarzyna Kruszka, Dawid Bielewicz, Jakub Dolata, Michal Szczesniak, Wojciech Karlowski, Artur Jarmolowski, Zofia Szweykowska-Kulinska

**Affiliations:** ^1^Department of Gene Expression, Institute of Molecular Biology and Biotechnology, Faculty of Biology, Adam Mickiewicz University in Poznan, Poznan, Poland; ^2^Department of Integrative Genomics, Institute of Anthropology, Faculty of Biology, Adam Mickiewicz University in Poznan, Poznan, Poland; ^3^Department of Computational Biology, Institute of Molecular Biology and Biotechnology, Faculty of Biology, Adam Mickiewicz University in Poznan, Poznan, Poland

**Keywords:** U12 introns, U2 introns, mRNA splicing, CBP20, *Arabidopsis thaliana*

In the original article, there was a mistake in both the figure and the legend for **Figure 6** as published. The western blot representing recognition of CBP20 in the wild type and various mutants of this gene in *A.thaliana*, is fused, because the original blots contained data from additional transgenic lines, which are not included in the paper. The data are from two independent western blots. All calculations concerning the amount of the CBP20 protein were carried out separately for each western blot. The correct figure and the legend for **Figure 6** appears below.

**Figure 6:**
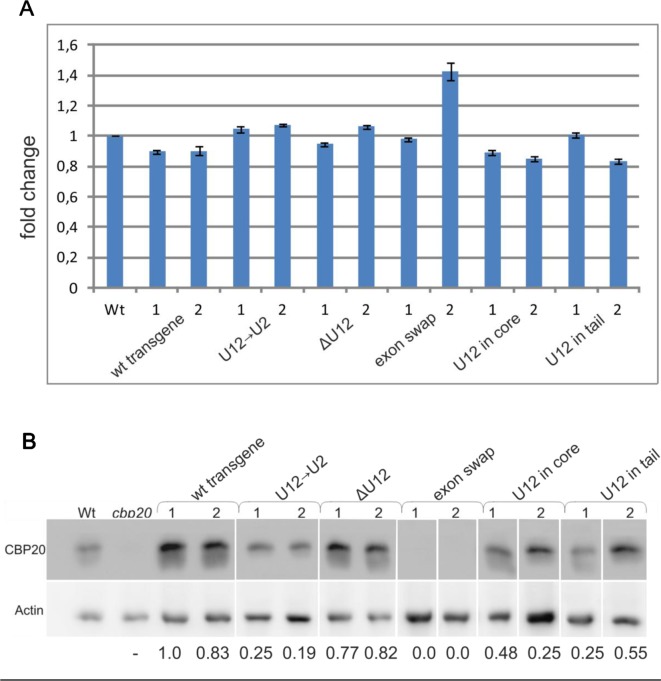
The localization, lack or replacement of the U12 intron in the *CBP20* gene does not influence the total level of the *CBP20* transcript but impacts the level of CBP20 protein in plants. **(A)** Real-time qPCR analysis of the total level of the *CBP20* transcript in maxi-gene transgenic lines. For each construct, two independent transgenic lines were analyzed. Wt – wild-type plants; *cbp20* – *cbp20* mutant line; wt transgene – wild-type *CBP20* gene structure; U12→U2 – *CBP20* gene in which the original U12 intron was replaced with the U2 intron derived from the *Arabidopsis CBP80* gene; ΔU12 – *CBP20* gene with U12 intron deletion; exon swap – the *CBP20* gene in which exons no. 4 and no. 5 flanking the U12 intron have been exchanged; U12 in core – a derivative of the U12→U2 construct in which the U12 intron has been introduced between exons no. 3 and no. 4; and U12 in tail – derivative of the U12→U2 construct in which the U12 intron has been introduced between exons no. 5 and no. 6. The *CBP20* mRNA level in Wt was taken as 1. Values are shown as the mean ± SD (*n* = 3) from three independent experiments. **(B)** Western blot analysis of CBP20 protein levels in transgenic *Arabidopsis* plants. For each construct, two independent transgenic lines were analyzed. Upper panel – immunoblot using antibodies against CBP20 protein; lower panel – immunoblot with antibodies against actin used as a loading control. Numbers below the western blot image are relative intensities of CBP20 bands calculated using the wt transgene (line 1) CBP20 level as 1. Lines are described as previously. The western blot representing recognition of CBP20 in wild type and various mutants of this gene in *A.thaliana* is fused because the original blots contained data from additional transgenic lines, not included in the paper. The data are from two independent western blots. All calculations concerning the amount of the CBP20 protein were carried out separately for each western.

The authors apologize for this error and state that this does not change the scientific conclusions of the article in any way. The original article has been updated.

