# Supplementary material for: A Role of U12 Intron in Proper Pre-mRNA Splicing of Plant Cap Binding Protein 20 Genes
Source: Front Plant Sci. 2018 Apr 16;9:475. doi: 10.3389/fpls.2018.00475 (PMC5932401; doi:10.3389/fpls.2018.00475)
Supplement: Supplementary file 1 [file Data_Sheet_1.docx]

Supplementary Material

**A role of U12 intron in Proper Pre-mRNA Splicing of Plant *Cap Binding Protein 20* Genes**

**Marcin Pieczynski^1^#, Katarzyna Kruszka^1^#, Dawid Bielewicz^1^, Jakub Dolata^1^, Michal Szczesniak^2^, Wojciech Karlowski^3^, Artur Jarmolowski^1^* and Zofia Szweykowska-Kulinska^1^***

1 Department of Gene Expression, Institute of Molecular Biology and Biotechnology, Faculty of Biology, Adam Mickiewicz University in Poznan, Poznan, Poland

2 Department of Integrative Genomics, Institute of Anthropology, Faculty of Biology, Adam Mickiewicz University in Poznan, Poznan, Poland

3 Department of Computational Biology, Institute of Molecular Biology and Biotechnology, Faculty of Biology, Adam Mickiewicz University in Poznan, Poznan, Poland

# These authors have contributed equally to this work

*Correspondence:

Artur Jarmolowski - artjarmo@amu.edu.pl

Zofia Szweykowska-Kulinska - zofszwey@amu.edu.pl

**Supplementary Figure S1.** Agarose gel electrophoresis of PCR products of *CBP20* cDNAs and genes from **(A)** *S. tuberosum* var. Sante (line 1) and Desiree (line 2), **(B)** *N. tabacum* var. Xanthi, **(C)** *H. vulgare* var. Sebastian and **(D)** *P. endiviifolia* subspecies B. M – DNA marker, K+ PCR positive control: actin cDNA amplification, K- PCR negative control.


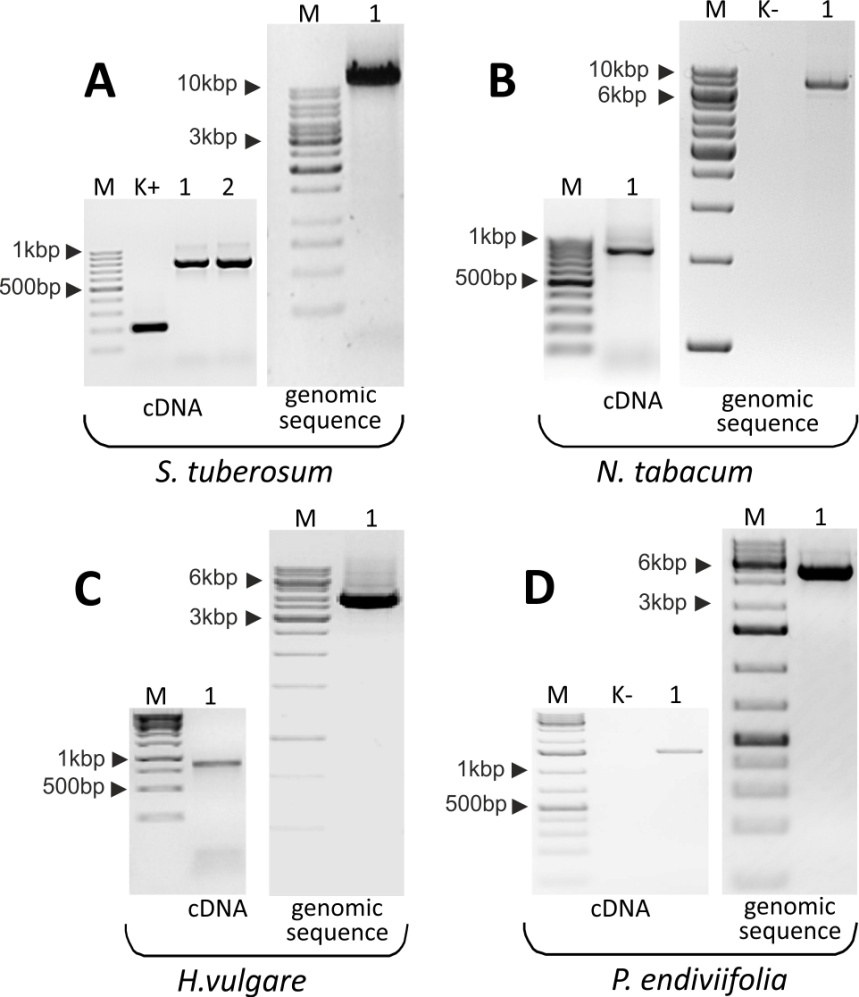


**Supplementary Figure S2.** A schematic diagram showing the consecutive steps of mini-gene construction. PCR-0 was performed only with constructs containing the U12 intron from the *S. tuberosum* or *V. vinifera CBP20* gene*.* Blue, red and green arrows depict PCR primers. The blue and red tails serve for the formation of a 40-bp overlap during PCR-1 and PCR-2 product assembly.

**
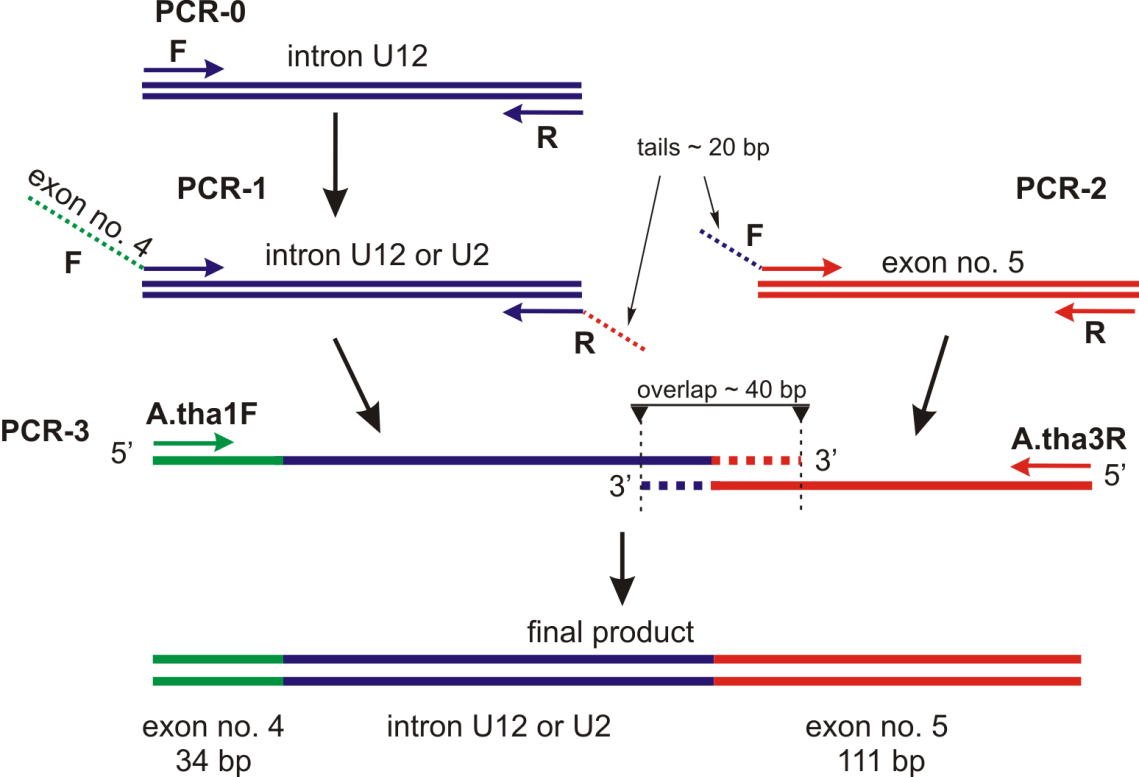
**

**Supplementary Figure S3.** A schematic diagram showing the consecutive steps of the maxi-gene construct preparation. Colored arrows depict PCR primers. Primer tails serve for the formation of an 40-bp overlap during PCR fragment assembly. Numbers 1-8 represent individual PCR products used for the assembly of the final maxi-gene construct (no. 9).


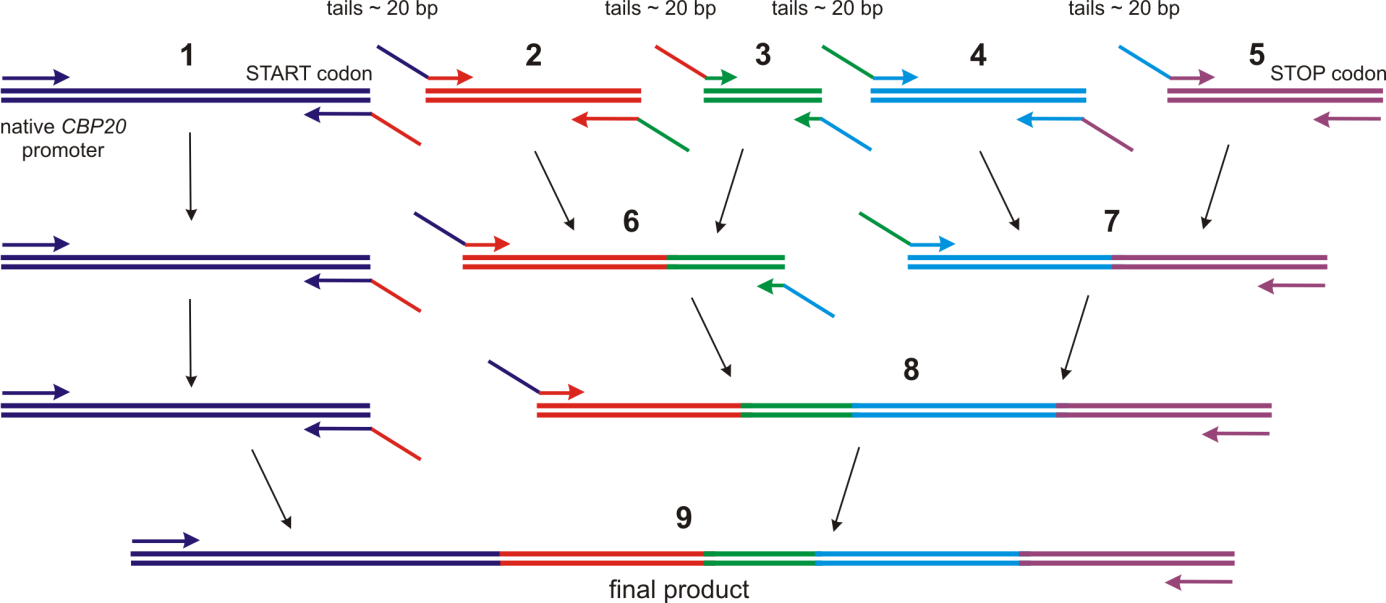


**Supplementary Figure S4.** CBP20 amino acid sequences alignment from five major groups of eukaryotes: protista (1), fungi (2), plants (3), invertebrates (4) and vertebrates (5). Amino acids involved in cap binding are marked in red. RRM domain is framed in blue, and NLS in the plant group is marked in green. Accessions used in the figure are listed below: *Plasmodium falciparum* - XP_001351463.1, *Plasmodium vivax* - XP_001615036.1, *Theileria annulata* - XP_953810.1, *Trypanosoma brucei* - XP_845318.1, *Trypanosoma cruzi* - XP_810208.1, *Leishmania braziliensis* - XP_001566695.1, *Dictyostelium* *discoideum* - XP_637361.1, *Tetrahymena thermophila* - XP_001033057.1, *Paramecium tetraurelia* - XP_001451519.1, *Thalassiosira pseudonana* - XP_002290373.1, *Hemiselmis andersenii* - XP_001712269.1, *Entamoeba dispar* - XP_001734617.1, *Ajellomyces capsulatus* - XP_001537919.1, *Coccidioides immitis* - XP_001247066.1, *Sclerotinia sclerotiorum* - XP_001585390.1, *Botryotinia fuckeliana* - XP_001557145.1, *Penicillium marneffei* - XP_002146544.1, *Talaromyces stipitatus* - XP_002478850.1, *Aspergillus fumigatus* - XP_755191.1, *Neosartorya fischeri* - XP_001260364.1 *Aspergillus niger* - XP_001395467.1, *Aspergillus flavus* - XP_002378107.1, *Penicillium chrysogenum* - XP_002558843.1, *Neurospora crassa* - XP_956595.1, *Podospora anserina* - XP_001907767.1, *Gibberella zeae* - XP_385664.1, *Phaeosphaeria nodorum* - XP_001799887.1, *Schizosaccharomyces pombe* - NP_596414.1, *Schizosaccharomyces japonicus* - XP_002172330.1, *Saccharomyces* *cerevisiae* - NP_015147.1, *Laccaria bicolor* - XP_001878240.1, *Nicotiana tabacum* - ACN43578.1, *Solanum tuberosum* - ACN43580.1, *Mirabilis jalapa* - AAT07459.1, *Populus trichocarpa* - XP_002309756.1, *Ricinus communis* - XP_002517864.1, *Vitis vinifera* - XP_002276868.1, *Arabidopsis thaliana* - NP_199233.1, *Lolium temulentum* - ACA24134.1, *Lolium perenne* - ABR26569.1, *Hordeum vulgare* - ACL83596.1, *Oryza sativa* - NP_001047412.1, *Zea mays* - NP_001150096.1, *Picea sitchensis* - ABK25999.1, *Physcomitrella patens* - Pp1s105_41V6.1, *Selaginella moellendorffii* - *Selaginella moellendorffii* v1.0 Scaffold 3333196:1, *Chlamydomonas reinhardtii* - XP_001690572.1, *Micromonas pusilla* - XP_003063843.1, *Micromonas* - XP_002505801.1, *Ostreococcus lucimarinus* - XP_001422671.1, *Branchiostoma floridae* - XP_002603437.1, *Strongylocentrotus purpuratus* - XP_802051.1, *Aedes aegypti* - XP_001657521.1, *Culex quinquefasciatus* - XP_001845223.1, *Anopheles* *gambiae* - XP_312392.3, *Pediculus humanus* - XP_002431557.1, *Apis mellifera* - XP_397316.1, *Nasonia vitripennis* - XP_001600470.2, *Tribolium castaneum* - XP_975066.2, *Drosophila melanogaster* - NP_524396.1, *Ixodes scapularis* - XP_002401570.1, *Acyrthosiphon pisum* - NP_001155582.1, *Caligus clemensi* - ACO14906.1, *Lepeophtheirus salmonis* - ACO11997.1, *Mus musculus* - EDK97761.1, *Rattus norvegicus* - EDM11451.1, *Homo sapiens* - NP_031388.2, *Monodelphis* *domestica* - XP_001371322.1, *Taeniopygia guttata* - XP_002188900.1, *Gallus gallus* - XP_422700.3, *Xenopus laevis* - CAA59259.1, *Esox lucius* - C1BY64.1, *Salmo salar* - C0H859.1, *Danio rerio* - NP_775356.1, *Siniperca chuatsi* - A2SW84.1, *Tetraodon nigroviridis* - CAF95006.1, *Ciona intestinalis* - XP_002127821.1, *Macaca mulatta* - XP_001091350.1, *Caenorhabditis elegans* - NP_001250552.1, *Caenorhabditis briggsae* - XP_002638958.1, *Brugia malayi* - XP_001892082.1, *Hydra* *magnipapillata* - XP_002160076.1, *Nematostella vectensis* - XP_001632015.1, *Trichoplax adhaerens* - XP_002107603.1.

**Supplementary Figure S5.** Intron no. 11 is spliced constitutively from its natural *CBP80* pre-mRNA. Upper panel – agarose gel electrophoretic separation of PCR products of the *CBP80* pre-mRNA fragment encompassing E11/E12 and E10/E11/E12/E13. Only fully spliced products were observed. Lower panel presents a schematic diagram for the performed RT-PCRs of *CBP80* pre-mRNA fragments. M - DNA length marker, K^-^ - PCR reaction negative control.


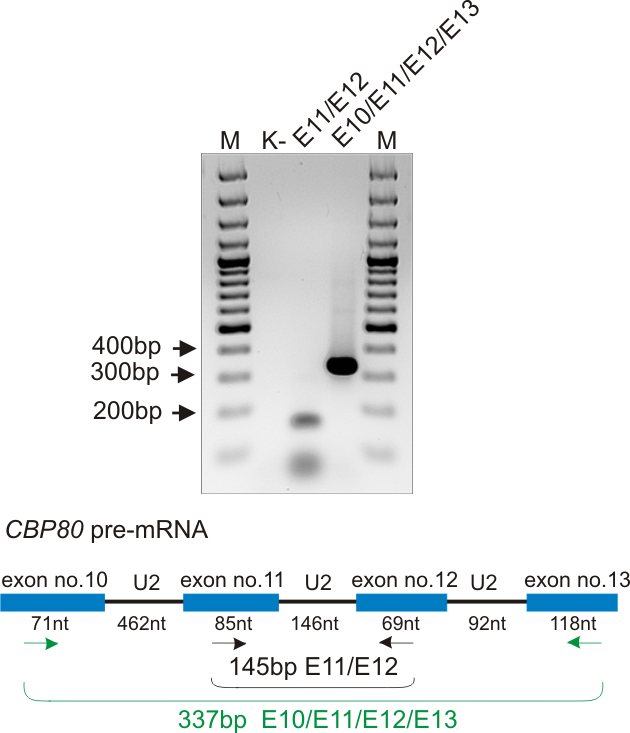


**Supplementary Figure S6.** Comparison of *CBP20* gene structures between plants and animals.

Green color depicts exons, yellow color marks U2 introns, and red color indicates U12 introns. E – exon, i – intron. Species used in this comparison – *Arabidopsis thaliana, Oryza sativa, Danio rerio, Xenopus tropicalis, Homo sapiens, Mus musculus, Gallus gallus,* and *Nematostella vectensis.*


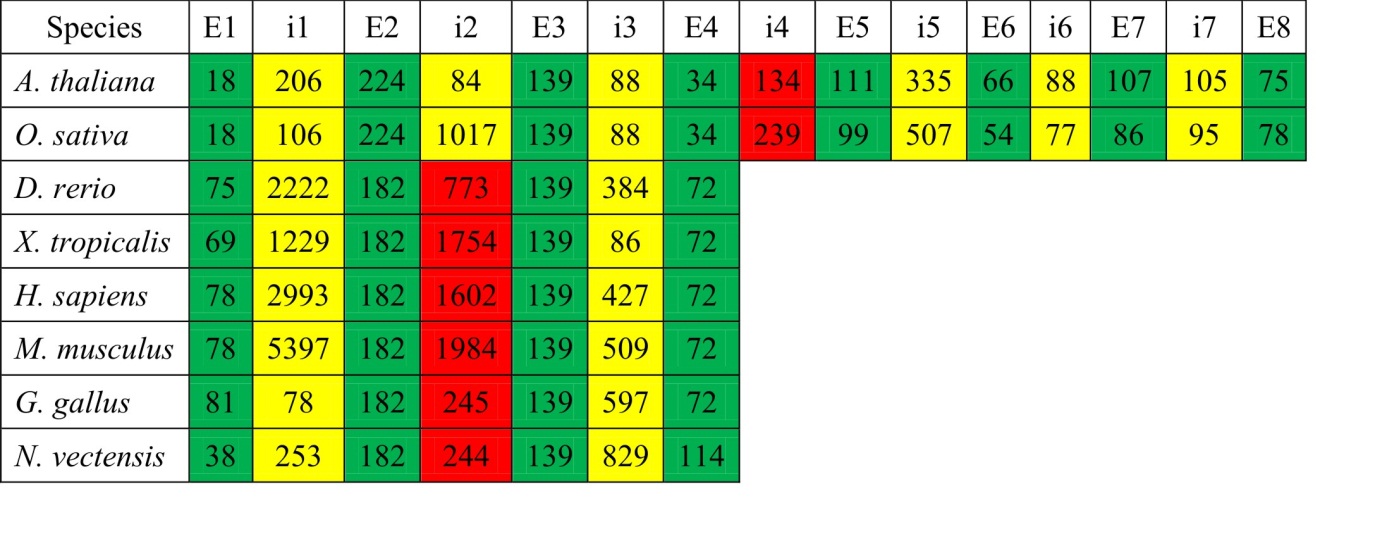


**Supplementary Figure S7.** Nucleotide sequence comparison between *P. patens* and *A. thaliana* of exon no. 4 (light gray) and exon no. 5 (dark gray) that surround the *CBP20* U12 intron. Deletion and insertion in *P. patens* are marked by blue squares.

*P. patens* GTGCGAGATGAGTACCGTACAGATTATGATCCTG/GGAGAGGAGGCTATGGAAAGTTGGTA

*A. thaliana* GTTCGTGATGAATACCGTACAGATTACGATCCTG/CTAGAGGTGGTTATGGGAAATTAGTT

** ** ***** ************** ******* ***** ** ***** ** ** **

*P. patens* CAGAAGGAGTTGGAAGTGCGGCGGCAGCTTGTGGAATATGACCAACCTGTGGGCATGGGA

*A. thaliana* CAGAAGGAACTCGAAGCACAAAGGCAGCTCGTGGATTACGGT---ACTGGCTCATTGGGA

******** * **** * ******* ***** ** * *** *****

*P. patens* GGGGTTCAATATCCT-AATCTGGGAGAAGGAGGTGGTGGAAATGAATTCAGGC

*A. thaliana* GC------TTATCCTCAAGCTGCGCCAACG--------------AATT-----

* ****** ** *** * ** * ****

**Supplementary Table S1.** Nucleotide sequences of primers used in this study

Primers used for *CBP20* intron sequences amplification from *S. tuberosum*, *N. tabacum* and *H. vulgare*.

| Intron number | *Solanum tuberosum* |
| --- | --- |
| 1 | Forward - 5’GATACGTAGTGGGAGATATCC 3’ Reverse - 5’GGAATTCTTATCCAATCCC 3’ |
| 2 | Forward - 5’CTTAGGATTTAGCCAAGATTTCAGC 3’ Reverse - 5’CTGGATCATAGTCGGTACGATATTCATC 3’ |
| 3 | Forward - 5’GTGGAACAATTCTTGATGATCGCCCTATTC3’ Reverse -5’CAACGATCCTGTACCATAATCCACTAG 3’ |
| 4 | Forward - 5’GTGGAACAATTCTTGATGATCGCCCTATTC 3’ Reverse - 5’CAACGATCCTGTACCATAATCCACTAG 3’ |
| 5 | Forward - 5’CTAGCAATACGTCGAGGTGGTTATGG 3’ Reverse - 5’GTCATAAGTCCTCTTTGGATAATCTG 3’ |
| 6 | Forward - 5’GTAGACATAATGGTGGAAACC 3’ Reverse - 5’CTAGATTCACGGTCATAAGTC 3’ |
| 7 | Forward - 5’CAGATTATCCAAAGAGGACTTATGAC 3’ Reverse - 5’GATACTTTCTTGGTGCAAATACGTTTC 3’ |
| Intron number | *Nicotiana tabacum* |
| 1 | Forward - 5’GAGCAAAACGTAATGGCTTC 3’ Reverse - 5’GGAATTCTTATCCAATCCC 3’ |
| 2 | Forward - 5’GAGCTGTTCTCTCGTGCTGG 3’ Reverse - 5’TGCCTACCTTCTTGAAATCC 3’ |
| 3 | Forward - 5’GTGGTAGGAGTGGTGGA 3’ Reverse - 5’CGTAGTCAGTACGATATTC 3’ |
| 4 | Forward - 5’GATGAATATCGTACCGACTATGATCCAG 3’ Reverse - 5’CAACGATCCTGTACCATAATCCACTAG 3’ |
| 5 | Forward - 5’CACAAAGGCAGCTAGTGGATTATGGTAC 3’ Reverse - 5’CATGTCGATAAGAGCCTCC 3’ |
| 6 | Forward - 5’GTTCTTATCGACACGGTAGAG 3’ Reverse - 5’CGAGATTCACGGTCGTAAC 3’ |
| 7 | Forward - 5’GACACAGGGAAGATGACCAC 3’ Reverse - 5’AGAGCAGGTCTTACAACTCTTTTC 3’ |
| Intron number | *Hordeum vulgare* |
| 1 | Forward - 5’AACAATGGCGTCCCTTTTCAAG 3’ Reverse - 5’TCGAGGCCCATGATGATCTTTT 3’ |
| 2 | Forward - 5’CTTCTACACCACGGAGGAGCA 3’ Reverse - 5’ATTGCCTGCCTTCTTCAAAGCC 3’ |
| 3 | Forward - 5’CTTTGAAGAAGGCAGGCAATGGG 3’ Reverse - 5’CATAATCCGTGCGGTACTCGTCTC 3’ |
| 4 | Forward - 5’CGAGTACCGCACGGATTATGATCCT 3’ Reverse - 5’TCTAGCTCTTTCTGAACCATCTTGCCA 3’ |
| 5 | Forward - 5’AGAGGTGGCTATGGCAAGATGG 3’ Reverse - 5’GTCATTCCGGTCACCATATCCTCTC 3’ |
| 6 | Forward - 5’GAGAGGATATGGTGACCGGAATGACAG 3’ Reverse - 5’TCTTAGAATCCGAGTCAGTTGCTCTCC 3’ |
| 7 | Forward - 5’TTACCAACGGAGACGATCAGCAC 3’ Reverse - 5’ATCAGAATCACCTTTCTCCCGAAACC 3’ |

Primers used for PCR amplification of *CBP20* cDNAs and genomic sequences.

| *CBP20* cDNA *Solanum tuberosum* | |
| --- | --- |
| StubCBP20F | 5’-GATACGTAGTGGGAGATATCCTTGGGAGC-3’ |
| StubCBP20R | 5’-CTAGGTACGCCGCTTCCTATCATCATCATC-3’ |
| *CBP20* cDNA *Nicotiana tabacum* | |
| NtabCBP20F | 5’-GAGCAAAACGTAATGGCTTC-3’ |
| NtabCBP20R | 5’-AGAGCAGGTCTTACAACTCTTTTC-3’ |
| *CBP20* cDNA *Hordeum vulgare* | |
| HvulCBP20F | 5’-CCCACAAGACCATCAAATTCC-3’ |
| HvulCBP20R | 5’-TTGGGCATTTGAATATGGCAG-3’ |
| *CBP20* cDNA *Pellia endiviifolia subspecies B* | |
| PendCBP20F | 5’- AAGCGAAGGGAAGTCGAATA -3’ |
| PendCBP20R | 5’- TTGGTGCCAAAATGATACTG -3’ |

Primers used for preparation of mini-gene constructs.

| Construct 1 – containing the U12 intron from *A. thaliana* | |
| --- | --- |
| Atha1F | 5’-TTTGGATCCGTTCGTGATGAATACCGTAC-3’ |
| Atha3R | 5’-TTTGGATCCAATTCGTTGGCGCAGCTTGAGG-3’ |
| Construct 2 – containing the U12 intron from *P. patens* | |
| Ppat1F | 5’-GGATCCGTTCGTGATGAATACCGTACAGATTACGATCC TGATATCCTTAACTGCTGTGC-3’ |
| Ppat2F | 5’-CCCTTAATTTAAAACAATTGCACCTAGAGGTGGTTATGGG-3’ |
| Ppat2R | 5’-CCCATAACCACCTCTAGGTGCAATTGTTTTAAATTAAGGG-3’ |
| Construct 3 – containing the U12 intron from *O. sativa* | |
| Osat1F | 5’-GGATCCGTTCGTGATGAATACCGTACAGATTACGATCCTGAT ATCCTTTGGTACTATTGAATTACC-3’ |
| Osat2F | 5’-GGCTACTTCCTTAATGAATCTTACCTAGAGGTGGTTATGGG-3’ |
| Osat2R | 5’-CCCATAACCACCTCTAGGTAAGATTCATTAAGGAAGTAGCC-3’ |
| Construct 4 – containing the U12 intron from *S. tuberosum* | |
| Stub1F | 5’-ATATCCTTCTCTTCTATCGATAAATTTGTG-3’ |
| Stub2R | 5’-ATTGCTAGTTAAGGTATTAAAAGATAGTCC-3’ |
| Stub1F | 5’-GGATCCGTTCGTGATGAATACCGTACAGATTACGATCCTGATA TCCTTCTCTTCTATCGATAAATTTGTG-3’ |
| Stub2F | 5’-GGACTATCTTTTAATACCTTAACTAGCAATACCTAGAGGTGGTTATGGG-3’ |
| Stub2R | 5’-CCCATAACCACCTCTAGGTATTGCTAGTTAAGGTATTAAAAGATAGTCC-3’ |
| Construct 5 – containing the U12 intron from *V. vinifera* | |
| Vvin1F | 5’-ATATCCTTTTGAATGTTTAATTTTGTG-3’ |
| Vvin2R | 5’-GTATAAACATTAAGGAAACTTCAAG-3’ |
| Vvin1F | 5’-GGATCCGTTCGTGATGAATACCGTACAGATTACGATCCTG ATATCCTTTTGAATGTTTAATTTTGTG-3’ |
| Vvin2F | 5’-CTTGAAGTTTCCTTAATGTTTATACCTAGAGGTGGTTATGGG-3’ |
| Vvin2R | 5’- CCCATAACCACCTCTAGGTATAAACATTAAGGAAACTTCAAG-3’ |
| Construct 6 – containing the U2 intron from *A. thaliana CBP80* gene* | |
| Atha1F | 5’-TTTGGATCCGTTCGTGATGAATACCGTAC-3’ |
| Atha3R | 5’-TTTGGATCCAATTCGTTGGCGCAGCTTGAGG-3’ |
| Construct 7 – containing the U2 intron from *P. sativum Legumin* gene | |
| Leg1F | 5’-GTTCGTGATGAATACCGTACAGATTACGATCCTGGTAAGTA ATAGTGTATCC-3’ |
| Leg2F | 5’-CGTAAATATGTGTATGCAGCTAGAGGTGGTTATGGG-3’ |
| Leg2R | 5’-CGTAAATATGTGTATGCAGCTAGAGGTGGTTATGGG-3’ |

* Sequence of mini-gene containing the U2 intron from *A. thaliana* *CBP80* gene was amplified using as a template the maxi-gene construct U12🡪U2, which contains the same U2 intron instead of the *CBP20* U12 intron.

Primers used for preparation of midi-gene constructs.

| Construct 8 – containing the *CBP20* gene fragment from *A. thaliana* | |
| --- | --- |
| AthF-long | 5’-TTTGGATCCGTTCTACTCTAGAGAGGATACTG-3’ |
| AthR-long | 5’-TTTGGATCCCTCCTCTTCCATGGCGATTTTGTCC-3’ |
| Construct 10 – containing the *CBP20* gene fragment from *P. patens* | |
| PpatF-long | 5’-TTTGGATCCATTCTATACAAGAGAAGATACTGAGGA -3’ |
| PpatR-long | 5’-TTTGGATCCCTCTATGTCGATTGCCTCCATTG -3’ |

Primers used for splicing analyses of mini- and midi-gene transcripts.

| zeinF | 5’-CCCAATTGTTCAACCCTAC-3’ |
| --- | --- |
| zeinR | 5’-GGTAAGATGCCTGTTGCGATTGC-3’ |
| zein3’-R | 5’-GGAAGAAATTGCTGGGGGTA-3’ |
| zeinF-FAM | 5’-6FAM-CCCAATTGTTCAACCCTAC-3’ |

Primers used for preparation of maxi-gene constructs.

| Prom-F | 5’-GGAGGGAACACCATAGTAGATTATGAAGC-3’ |
| --- | --- |
| Stop-R | 5’-TTAAGATCTTCTCTTCCGATCATCTTCACC-3’ |
| Prom-NotI-F | 5’-TTTGCGGCCGCGGAGGGAACACCATAGTAGATTATGAAGC-3’ |
| Stop-NotI-R | 5’-TTTGCGGCCGCTTAAGATCTTCTCTTCCGATCATCTTCACC-3’ |
| 2-1F | 5’-CGTACAGATTACGATCCTGGTAACTTCTGTTGTATAAATTTTGG-3’ |
| 2-1R | 5’-CCAAAATTTATACAACAGAAGTTACCAGGATCGTAATCTGTACG-3’ |
| 2-2F | 5’-CAATTGTATCTACTGTTTTTCAGCTAGAGGTGGTTATGGG-3’ |
| 2-2R | 5’-CCCATAACCACCTCTAGCTGAAAAACAGTAGATACAATTG-3’ |
| 3-1F | 5’-CCGTACAGATTACGATCCTGCTAGAGGTGGTTATGGG-3’ |
| 3-1R | 5’-CCCATAACCACCTCTAGCAGGATCGTAATCTGTACGG-3’ |
| 4-1F | 5’-GGTTTTCTAATTTCATACAGCTAGAGGTGGTTATGGG-3’ |
| 4-1R | 5’-CCCATAACCACCTCTAGCTGTATGAAATTAGAAAACC-3’ |
| 4-2F | 5’-CCTCAAGCTGCGCCAACGAATTATATCCTTTTAGTCCGCATAG-3’ |
| 4-2R | 5’-CTATGCGGACTAAAAGGATATAATTCGTTGGCGCAGCTTGAGG-3’ |
| 4-3F | 5’-CCTTAACTCATTGATTACGTTCGTGATGAATACCGTAC-3’ |
| 4-3R | 5’-GTACGGTATTCATCACGAACGTAATCAATGAGTTAAGG-3’ |
| 4-4F | 5’-CCGTACAGATTACGATCCTGGTATGTTTTTTTCAACTTTCTACC-3’ |
| 4-4R | 5’-GGTAGAAAGTTGAAAAAAACATACCAGGATCGTAATCTGTACGG-3’ |
| 5-1F | 5’-GTGGTAGAAGCGGTGGCCAGATATCCTTTTAGTCCGCATAG-3’ |
| 5-1R | 5’-CTATGCGGACTAAAAGGATATCTGGCCACCGCTTCTACCAC-3’ |
| 6-1F | 5’-CCTTAACTCATTGATTACATGGAAATGGAAGGCGTGG-3’ |
| 6-1R | 5’-CCACGCCTTCCATTTCCATGTAATCAATGAGTTAAGG-3’ |
| egz-4 | 5’-GTTCGTGATGAATACCGTACAGATTACGATCCTG-3’ |

Primers used for splicing analyses of maxi-gene transcripts.

| Splice-CBP20F | 5’-GGGATTCCAAGAAGGAAGACA-3’ |
| --- | --- |
| Splice-CBP20R | 5’-TTTGTCCTCCTTGACCATAGTTTC-3’ |
| Splice-CBP20F-FAM | 5’-6FAM-GGGATTCCAAGAAGGAAGACA-3’ |
| Actin-Ath-F | 5’-CTCGGCCTTGGAGATCCACA-3’ |
| Actin-Ath-R | 5’-ACATTGTGCTCAGTGG-3’ |
| F_cbp20_end | 5’-CAAACATGCCACTTTCATGC-3’ |
| R_cbp20_end | 5’-AGCCAAAGTGATGATGCAGA-3’ |
| EF1a For | 5’ GACATGAGCCAGACTGTTGCA-3’ |
| EF1A Rev | 5’- CCGGTTGGGTCCTTCTTGT-3’ |

Primers used for splicing analyses of artificial *CBP20* gene transcripts.

| Splice1-CBP80F | 5’-ACGAGTGTGCATCCTACATGG-3’ |
| --- | --- |
| Splice1-CBP80R | 5’-CAAAGATCCATAATCACGAGTGT-3’ |
| Splice2-CBP80F | 5’-AACCAATTGATCGTTTTGTCG-3’ |
| Splice2-CBP80R | 5’-AAGTGGTGAGAAAACCAGAGGA-3’ |

**Supplementary Table S2**. Quantitative Real-time PCR data used for calculations of the mini-gene transcript splicing efficiency (**Figure 2C**). The qRT experiment was performed using the absolute quantification method. The quantity of spliced and unspliced molecules was calculated based on standard curves determined independently for spliced and unspliced transcripts. Values are shown as the mean from three independent experiments (n=3). SD – the standard deviation.

| Construct no. | Spliced transcript | | | Unspliced transcript | | |
| --- | --- | --- | --- | --- | --- | --- |
|  | Mean Ct value | Mean molecule quantity | SD | Mean Ct value | Mean molecule quantity | SD |
| Construct 1  *A. thaliana* | 25.71 | 93.68 | 15.16 | 17.93 | 104071.54 | 16451.13 |
| Construct 2  *P. patens* | 26.05 | 74.32 | 13.82 | 17.84 | 397188.78 | 42558.64 |
| Construct 3  *O. sativa* | 22.65 | 767.78 | 126.69 | 20.87 | 662838.25 | 46314.61 |
| Construct 4  *S. tuberosum* | 22.12 | 1100.41 | 23.10 | 19.08 | 186904.31 | 73280.55 |
| Construct 5  *V. vinifera* | 21.26 | 1992.59 | 68.28 | 21.11 | 62282.78 | 6428.19 |
